# Rising Income Inequality and the Impact of r-g in the United States

**DOI:** 10.1111/roiw.70000

**Published:** 2025-02-18

**Authors:** Antonio Abatemarco, Elena Lagomarsino, Alessandro Spiganti

**Affiliations:** 1 Department of Economics and Statistics, University of Salerno, Salerno, Italy; 2 Department of Economics, University of Genoa, Genoa, Italy; 3 Department of Economics, Ca’ Foscari University of Venice, Venice, Italy

**Keywords:** factor decomposition, income distribution, inequality dynamics, PSID, r-g gap, D31, D33, J31

## Abstract

This paper proposes a novel non-parametric strategy to test ‘Piketty’s third law,’ postulating that a positive gap between the rate of return on capital (r) and the economy’s growth rate (g) is associated with increased income inequality. The strategy is based on a decomposition of inequality changes over time that allows investigating the existence of a distributive effect of the r-g gap and its relative importance with respect to other drivers of income inequality changes over time. Applying this strategy to data from the Panel Study of Income Dynamics on the last 50 years, we find support for the existence of Piketty’s law in the evolution of income inequality in the United States. Whereas factors other than Piketty’s income dynamics predominantly drive overall inequality, the r-g gap accounts for approximately 10% of the trend in income inequality in the medium- and long-term.

## 1 | Introduction

Piketty (2014) introduced two fundamental laws of capitalism, which together suggest an inverse relationship between the share of capital in national income and the growth rate of the economy. Coupled with the tendency for the rate of return on capital r to exceed the growth rate of national income g, this leads to increasing capital-to-income ratios. Consequently Piketty (2014), argues that a positive r-g gap will result in higher inequality: since capital ownership is concentrated among richer individuals, a faster growth of capital relative to labor income results in a capital-driven rise in overall income (and, over time, wealth) inequality. This relationship between the r-g gap and inequality has come to be known as ‘Piketty’s third general law’ (Acemoglu and Robinson 2015).

However, empirical studies have so far failed to identify a significant and positive relationship between the r-g gap and changes in income inequality (e.g. Roine, Vlachos, and Waldenström 2009; Acemoglu and Robinson 2015; Góes 2016).^1^ We argue that this is not surprising, as these attempts disregard the complexity of inequality dynamics, which are shaped by the evolution of many different factors. On the one hand, shifts in income inequality over time may stem from changes in the covariance between income sources, such as capital and labor. For example, inequality tends to rise when capital income is both concentrated among top earners and grows faster than labor income. On the other one, inequality may be driven by changes in the distribution of each income source independently. For example, the labor income distribution may become more concentrated at the top due to skill-biased technological change, while the capital income distribution may experience a similar trend due to increasing corporate retained earnings or the growing market power of firms.

Building on this intuition, we propose a non-parametric strategy to empirically test Piketty’s third law based on the decomposition of changes in income inequality over time. Using a counterfactual approach, we are able to identify the impact of Piketty’s prediction on the observed dynamics of the income distribution when other inequality drivers are also accounted for. More specifically, we decompose income inequality changes over time into a ‘Piketty’s component’–which captures the variation in inequality that would have occurred if labor and capital incomes had simply grown at their respective rates, holding other factors constant–and a ‘non-Piketty’s component’–a residual term accounting for all other potential sources of changes in inequality.

Furthermore, we outline an empirical method to evaluate Piketty’s third law, applying the inequality decomposition to microeconomic data. This approach involves two steps. First, researchers should test an existence condition, requiring Piketty’s component to exhibit the same sign as the r-g gap. Second, if this condition holds, they can then assess the relative importance of Piketty’s component in explaining changes in overall income inequality compared to other contributing factors.

We emphasize that Piketty’s third law cannot be tested solely by examining the evolution of overall income inequality. Even if the existence condition is satisfied, income inequality may align with or diverge from the sign of the r-g gap, depending on whether the non-Piketty component exerts a dominant influence. Conversely, even if the existence condition is not met, overall inequality could still evolve in a way that appears consistent with Piketty’s third law, but this alignment would be entirely driven by factors unrelated to the r-g gap.

A by-product of our non-parametric strategy is the possibility to further investigate the determinants of the non-Piketty’s component, which becomes particularly important if this component is the main responsible for the observed dynamics of income inequality. By applying standard decomposition techniques proposed by Atkinson (2009) to our dynamic setting, we decompose the non-Piketty’s component into between-source and within-source variations. The former captures changes in the covariance between labor and capital incomes: for instance, we might observe an increase in the non-Piketty’s component if capital income becomes more positively correlated with labor income over time, such as when the richest individuals earn high income from both sources. Conversely, the latter reflects changes in the distributions of each income source taken separately; for example, if the distribution of one or both sources becomes more concentrated at the top, this would contribute to increasing the non-Piketty’s component.

In the second part of our paper, we apply the illustrated strategy to analyze the evolution of income inequality in the United States over the past 50 years, using data from the Panel Study of Income Dynamics (PSID). Our findings support the existence of Piketty’s third law, as the sign of the r-g gap consistently aligns with the direction of Piketty’s component. Moreover, we find that Piketty’s component accounts for approximately 10% of the observed long-run changes in income inequality. In other words, factors other than r-g have been the predominant drivers of the observed dynamics of the income distribution in the past 50 years in the United States, but we do find a relevant impact of Piketty’s component in the long run, which, in our view, motivates its inclusion among the drivers of income inequality changes. Finally, among predominant factors, we find the contribution of the within-source variation, which explains over 90% of the non-Piketty’s component. This result is consistent with the view that increased wages at the top of the income distribution, driven by factors like skill-biased technological change and increasing top managerial compensation, have been significant contributors to rising inequality in the last 50 years.

All in-all, our contribution is twofold: methodological and empirical. Methodologically, we propose a decomposition of inequality changes over time by which the effect of the r-g gap on the dynamics of the income distribution can be measured by using standard non-parametric estimation tools. In this framework, we are able to disentangle the existence of a distributive impact of the r-g gap from its relative contribution with respect to other drivers of income inequality. Empirically, we test the validity of Piketty’s third law within a microeconomic setting by using PSID data. Our findings suggest that a distributive impact of the r-g gap does exists, even if this is not the dominating factor among other inequality drivers, especially in the short run.

The remainder of this paper is organized as follows. Section 2 briefly contextualizes Piketty’s third law before justifying the need for considering different determinants of inequality in a test of this law. Section 3 explains our proposed methodology. Section 4 describes the data used, the results, and some robustness checks. Finally, Section 5 concludes.

## 2 | Piketty’s Hypothesis

Piketty (2014) starts by introducing two ‘fundamental laws of capitalism’. The first one is simply a definition, α=r(K/Y), where α is the capital share (i.e., the share of national income distributed as capital income), r is the net real rate of return on capital (or the real interest rate), K is the capital stock, and Y is national income or gross domestic product (GDP). The second law, which readily follows from the steady state of the Harrod-Domar-Solow’s model of economic growth (but see e.g. Krusell and Smith 2015), states that the capital-to-income ratio K/Y converges in the long run to s/g, where s is the saving rate and g is the growth rate of the economy: intuitively, the accumulated capital stock becomes relatively more important in a society with a low growth rate. The combination of these two laws leads to α=r(s/g). Therefore, if r and s are approximately constant (as posited by Piketty 2014), the first general law (as defined by Acemoglu and Robinson 2015) follows: there is an inverse relationship between the share of capital in national income and the growth rate of the economy.

Piketty (2014) second general law is the tendency for returns to capital to grow faster than national income, represented by the well-known equation r>g. Since growth rates have usually been lower than rates of returns until the 19th century, the combination of the first and second general laws explains historically high capital-to-income ratios; likewise, the fact that growth rates were exceptionally high and rates of return exceptionally low during the 20th century explains why these ratios did not return to pre-WWI levels in the postwar period. However, this tendency has since reasserted itself, and Piketty and Saez (2014) maintain that r-g will likely increase in the 21st century, insofar as population and productivity growth will slow down and that rates of return to capital will rise.

While the resulting high capital-to-income ratio (or, equivalently, capital share) is not problematic per se Piketty (2014), it argues that capital ownership tends to be much more concentrated than labor income. Piketty’s third general law follows: when returns to capital grow faster than national income (i.e., r>g), existing inequalities in capital ownership will tend to be amplified. This occurs because national income (and thus labor income) increases at the rate of growth g, whereas capital accumulates at the rate of interest r. Although Piketty (2014) mechanism primarily addresses inequality in capital ownership (and thus in wealth), a positive r-g gap could also amplify income inequality. This happens as individuals in top income classes typically rely less on labor income and more on returns from accumulated capital. In other words, given the unequal distribution of both capital ownership and income, a positive r-g gap could represent a ‘fundamental force of divergence’ (Piketty 2014) resulting in a capital-driven increase in income inequality.

It should be noted that, following from this third law, a major explanation for rising income inequality may lie in the evolution of the composition of income in terms of sources over time. As underlined by Atkinson (1997, 2009), this introduces a link between the personal and functional (that is, the share of total income going to the factors of production) income distributions, which has seen a revival of interest in the economic literature following Piketty (2014). Indeed, if capital is held by a small fraction of the population with overall high income, a positive r-g gap would lead to rising capital share and between-source inequality, and thus to greater overall inequality. However, the empirical link between greater capital share and increased inequality is not unambiguous (e.g. Jacobson and Occhino 2012; Francese and Mulas-Granados 2015; Milanović 2017; Bengtsson and Waldenström 2018; Ranaldi 2022; Ranaldi and Milanović 2022; Iacono and Ranaldi 2023).

Formal parametric testings, regressing different income inequality measures on the r-g gap, have generally failed to find significant evidence of a positive relationship (e.g. Roine, Vlachos, and Waldenström 2009; Acemoglu and Robinson 2015; Góes 2016). However, we argue that, even if Piketty’s third general law were to hold, it does not necessarily imply that it is verified whenever income inequality is found to increase during periods with a positive r-g gap; equivalently, since the dynamics of income inequality may be driven by the impact of other determinants, Piketty’s third law may still be at play even when the observed pattern of income inequality is not consistent with the r-g gap. Indeed, as underlined by Piketty (2015) himself, r-g is not the only or even the primary mechanism determining the evolution of income inequality.

In our framework, two drivers of inequality other than the one predicted by Piketty’s third law are accounted for. On the one hand, when total income is the sum of incomes from different sources, the observed income inequality dynamics may be due to the way these different income sources covariate with each other over time. Indeed, if the covariance between capital income and labor income increases over time, individuals earning higher labor income are increasingly likely to also receive higher capital income (a phenomenon that Milanović 2019, calls homoploutia). This dynamic tends to create polarization in the overall income distribution and, thus, to increased inequality, pushing more income toward the top of the distribution and leaving the bottom relatively stagnant. Indeed Berman and Milanović (2024), document a sharp increase in the share of top decile capital income earners in the top decile of labor income earners in the United States since 1985, which may account for up to 20% of the increase in interpersonal income inequality.^2^

On the other hand, it may be the case that the main driver of inequality is to be found in the distribution of one single income source, which may have changed over time. In other words, inequality patterns may be mostly affected by the dynamics of *within-source* inequality, that is, by the evolution of labor and capital income distributions taken separately. For example, it is commonly argued that increasing income inequality towards the end of the 20th century has mostly been driven by a dramatic increase in wages at the top of the income distribution (Piketty and Saez 2003), due to e.g., skill-biased technological change (Card and DiNardo 2002; Autor, Katz, and Kearney 2006), exploding top managerial compensation (Piketty 2015), and large cuts in top tax rates (Piketty, Saez, and Stantcheva 2014). Conversely, rising income inequality in the 21st century may owe primarily to an upsurge in capital income, due to a rise in corporate retained earnings (Piketty, Saez, and Zucman 2018) or in firms’ monopoly power (Stiglitz 2016).

Provided that within-source and between-source determinants may sensibly affect the dynamics of overall income inequality, in what follows we propose a measurement strategy that allows to test and to assess the relative impact of Piketty’s third law when the other drivers of inequality are accounted for.

## 3 | Methodology

### 3.1 | Preliminaries

Let $Y_{t}≔\left\{y_{1,t},\ldots ,y_{n_{t},t}\right\}\in R^{n_{t}}$, with $y_{1,t}\leq \ldots \leq y_{n_{t},t}$, be the *factual* ordered income distribution in a population of $\left\{1,\ldots ,n_{t}\right\}$ individuals at time t. We consider two possible sources of income: labor income $Y_{t}^{w}≔\left\{y_{1,t}^{w},\ldots ,y_{n_{t},t}^{w}\right\}\in R_{\geq 0}^{n_{t}}$ and capital income $Y_{t}^{c}≔\left\{y_{1,t}^{c},\ldots ,y_{n_{t},t}^{c}\right\}\in R^{n_{t}}$, so that $y_{i,t}=y_{i,t}^{w}+y_{i,t}^{c}\;\forall i$.

Using $g_{t}$ and $r_{t}$ to indicate the real growth rates between t and t+k of labor and capital income, respectively, we let ${\overset{ˆ}{Y}}_{t+k|t}≔\left\{{\overset{ˆ}{y}}_{1,t+k|t},\ldots ,{\overset{ˆ}{y}}_{n_{t+k|t},t+k|t}\right\}\in R^{n_{t+k|t}}$ be the *counterfactual* income distribution at t+k one would observe if labor and capital income grew at their respective rate between t and t+k, that is,

(1) $${\overset{ˆ}{y}}_{i,t+k|t}≔\left(1+g_{t}\right)y_{i,t}^{w}+\left(1+r_{t}\right)y_{i,t}^{c}\;\forall i$$

Hereafter, we refer to this counterfactual distribution as ‘Piketty’s income dynamics’; notably, such dynamics exclude any source of income variations other than differences in the growth rates $g_{t}$ and $r_{t}$.

For Piketty’s third law to hold, Piketty’s income dynamics (i.e., from $Y_{t}$ to ${\overset{ˆ}{Y}}_{t+k|t}$) must be inequality increasing (cfr., decreasing) in time intervals with a positive (cfr., negative) r-g gap, which we refer to as ‘existence condition’. For this to happen, it must be the case that capital income is mostly held by top income classes (see also Atkinson 2009; Milanović 2017). In other words, for Piketty’s income dynamics to generate inequality changes in the same direction as the one predicted by Piketty’s third law, if $r_{t}>g_{t}$ (cfr. $r_{t}<g_{t}$), the Lorenz curve of the factual distribution must lie nowhere below (cfr. above) the one of the counterfactual distribution, or, equivalently, the concentration curve of labor income must lie nowhere below the concentration curve of capital income when both distributions are ranked by overall income.^3^ For Piketty’s third law to exist, distributive conditions must hold in the decomposition by income sources.

Provided that such existence condition is satisfied, one can then assess the relative importance of Piketty’s income dynamics in explaining the evolution of income inequality over time. Given the Lorenz curves $L_{t+k}(p)$ and ${\overset{ˆ}{L}}_{t+k|t}(p)$ of, respectively, the factual and counterfactual income distribution at time t+k, such relative importance can be measured by the share of the overall movement in the factual Lorenz curves, that is, $\left|L_{t+k}(p)-L_{t}(p)\right|$, that is determined by the contribution of Piketty’s income dynamics, that is, $\left|{\overset{ˆ}{L}}_{t+k|t}(p)-L_{t}(p)\right|$, as opposed to the contribution of its complement, that is, $\left|L_{t+k}(p)-{\overset{ˆ}{L}}_{t+k|t}(p)\right|$. In case of conflict between Piketty and non-Piketty inequality drivers, the dynamics of the factual income distribution will be consistent with the one predicted by Piketty’s third law if and only if the Piketty’s component is greater in magnitude than its complement, that is, if $\left|{\overset{ˆ}{L}}_{t+k|t}(p)-L_{t}(p)\right|>\left|L_{t+k}(p)-{\overset{ˆ}{L}}_{t+k|t}(p)\right|\;\forall \;p$.

Indeed, income inequality patterns between the factual income distributions $Y_{t}$ and $Y_{t+k}$ are not uniquely determined by the evolution of the r-g gap, as other factors may contribute to, and occasionally drive, observed changes in inequality. As a consequence, Piketty’s third law cannot be tested by simply considering the information in the transition from $Y_{t}$ to $Y_{t+k}$, as non-Piketty inequality drivers may predominantly affect the overall dynamics of the income distribution. In such a case, even if the existence condition holds, the factual income distribution may still move over time in a direction opposite to the one predicted by Piketty’s income dynamics; conversely, even if the factual income distribution moves in the direction predicted by Piketty’s third law, it may be the case that the existence condition is actually violated.

### 3.2 | Decomposition of Inequality Changes

Here, we reformulate the previous discussion in (the more robust) general partial ordering approach to inequality (i.e., through Lorenz and concentration curves) using a complete ordering approach by considering any inequality index satisfying the well-known Pigou-Dalton principle of transfer (Hardy, Littlewood, and Polya 1929). Indeed, this allows testing the validity of Piketty’s third law with microeconomic data and quantifying its relative importance on overall income inequality dynamics when accounting for the other drivers of inequality.

In the field of non-parametric estimation, several decomposition procedures have been proposed based on generalized entropy (GE) measures (Shorrocks 1982), Gini index (Lerman and Yitzhaki 1985), and, more generally, any inequality index through the implementation of Shapley’s rule (Shorrocks 2013). In this paper, we consider the generalized entropy index GE(2),^4^ which is equal to half the squared coefficient of variation (CV),

(2) $$GE(2)=\frac{CV^{2}}{2}=\frac{\sigma_{Y}^{2}}{2\mu_{Y}^{2}}$$

where $\mu_{Y}$ indicates mean income and $\sigma_{Y}^{2}$ is total income variance. Henceforth, we omit the parameter from GE(2) for ease of reading.

After establishing an approach to measure inequality, we proceed to present a novel methodology to decompose income inequality changes. Within a dynamic setting, income inequality changes between any two periods can be decomposed as

(3) $$\Delta GE_{t+k}=GE_{t+k}-GE_{t}\;=\left(GE_{t+k}-{\hat{GE}}_{t+k|t}\right)+\left({\hat{GE}}_{t+k|t}-GE_{t}\right)\;=\Delta GE_{t+k}^{NP}+\Delta GE_{t+k}^{P}$$

where ${\hat{GE}}_{t+k|t}$ measures inequality in the counterfactual distribution ${\overset{ˆ}{Y}}_{t+k|t}$. As a consequence, $\Delta GE_{t+k}^{P}$ is the change in the inequality index between t and t+k that one would have observed if labor and capital incomes at t had simply grown at their respective rates, without any change in their underlying distributions. Thus, $\Delta GE_{t+k}^{P}$-that we name *Piketty’s component* – is a measure of the expected effect of Piketty’s income dynamics on the overall change in income inequality, whereas $\Delta GE_{t+k}^{NP}$ – that we name *non-Piketty’s component* – represents the effect of all other potential drivers of inequality.^5^

Exploiting the proposed decomposition in Equation (3), we can then test the existence condition of Piketty’s third law by simply checking if $\Delta GE_{t+k}^{P}$ shows a positive (cfr. negative) value in periods with $r_{t}>g_{t}$ (cfr. $r_{t}<g_{t}$). Furthermore, we can measure its relative importance through the assessment of the relative contribution of $\Delta GE_{t+k}^{P}$ to the observed dynamics of overall income inequality $\Delta GE_{t+k}$.

Additionally, this decomposition allows for an even deeper investigation of income inequality. In particular, we can further decompose the non-Piketty’s component to try to understand its origin. To this aim, provided that $Y=Y^{w}+Y^{c}$, recall from Shorrocks (1982) that GE is decomposable by income sources as follows,

(4) $$GE=\frac{1}{2}\left[\frac{\sigma_{\left(Y,Y^{w}\right)}}{\mu_{Y}^{2}}+\frac{\sigma_{\left(Y,Y^{c}\right)}}{\mu_{Y}^{2}}\right]$$

where $\sigma_{\left(Y,Y^{w}\right)}$ and $\sigma_{\left(Y,Y^{c}\right)}$ are the covariances between total income and income from labor and capital, respectively, so that the two components on the right-hand side of equation (4) measure the contribution of labor and capital income to overall income inequality, respectively. Equation (4) is particularly convenient in that it highlights the role of the covariance between a single income source and overall income, which is immediately relevant and intelligible for our purposes.^6^

In addition, as shown in Atkinson (2009), covariance properties allow to decompose GE in Equation (4) as

(5) $$GE=\frac{\sigma_{\left(Y^{w},Y^{c}\right)}}{\mu_{Y}^{2}}+\frac{\sigma_{Y^{w}}^{2}+\sigma_{Y^{c}}^{2}}{2\mu_{Y}^{2}}\;=\left(\frac{\sigma_{\left(Y^{w},Y^{c}\right)}}{\mu_{Y}^{2}}\right)+\left(\frac{\mu_{Y^{w}}^{2}}{\mu_{Y}^{2}}GE^{w}+\frac{\mu_{Y^{c}}^{2}}{\mu_{Y}^{2}}GE^{c}\right)\;=BS+WS$$

where $\mu_{Y^{w}},\mu_{Y^{c}},\sigma_{Y^{w}}^{2}$, and $\sigma_{Y^{c}}^{2}$ are the means and the variances of income from labor and capital, $\sigma_{\left(Y^{w},Y^{c}\right)}$ is the covariance between labor income and capital income, and $GE^{w}$ and $GE^{c}$ are half the squared coefficients of variation calculated on income from the two sources in isolation. Within a given period, the first term on the right-hand side of equation (5) quantifies the contribution of *between-source* inequality to overall income inequality, which we define as BS, whereas the second component measures the contribution of *within-source* inequality, which we define as WS. The second line underlines that this latter component can be further decomposed to highlight the contribution of the variability of each income source taken separately, weighted by the square of the income share. Intuitively, provided that capital and labor income are unequally distributed (that is, WS>0), overall inequality is greater when top capital income owners are also top labor income recipients (i.e., BS>0).

Then, combining Equations (3) and (5), we obtain

(6) $$\Delta GE_{t+k}^{NP}=GE_{t+k}-{\hat{GE}}_{t+k|t}\;=\left(BS_{t+k}-{\hat{BS}}_{t+k|t}\right)+\left(WS_{t+k}-{\hat{WS}}_{t+k|t}\right)\;=\Delta {\hat{BS}}_{t+k}^{NP}+\Delta {\hat{WS}}_{t+k}^{NP}$$

where ${\hat{BS}}_{t+k|t}$ and ${\hat{WS}}_{t+k|t}$ describe the contribution of between-source and within-source inequality in the counterfactual distribution ${\overset{ˆ}{Y}}_{t+k|t}$, respectively. Therefore, $\Delta {\hat{BS}}_{t+k}^{NP}$ and $\Delta {\hat{WS}}_{t+k}^{NP}$ identify, respectively, the contribution of between-source and within-source inequality to overall income dynamics, net of Piketty’s income dynamics.

Finally, by recalling the definition of WS in Equation (5), $\Delta {\hat{WS}}_{t+k}^{NP}$ can be further decomposed into

(7) $$\Delta {\hat{WS}}_{t+k}^{NP}=\left(\frac{\mu_{Y_{t+k}^{w}}^{2}}{\mu_{Y_{t+k}}^{2}}GE_{t+k}^{w}-\frac{\mu_{Y_{t+k|t}^{w}}^{2}}{\mu_{Y_{t+k|t}}^{2}}GE_{t+k|t}^{w}\right)+\left(\frac{\mu_{Y_{t+k}^{c}}^{2}}{\mu_{Y_{t+k}}^{2}}GE_{t+k}^{c}-\frac{\mu_{Y_{t+k|t}^{c}}^{2}}{\mu_{Y_{t+k|t}}^{2}}GE_{t+k|t}^{c}\right)\;=\Delta {\hat{WS}}_{t+k}^{NP\left(w\right)}+\Delta {\hat{WS}}_{t+k}^{NP\left(c\right)}$$

where $\Delta {\hat{WS}}_{t+k}^{NP(s)}$ measures the contribution of income source s={w,c} to the contribution of within-source inequality.

## 4 | Empirical Analysis

### 4.1 | Data

We use data from the Panel Study of Income Dynamics (PSID). Panel study of income dynamics (2024), the world’s longest-running household panel survey: it started in 1968, with over 18,000 individuals living in 5,000 families in the United States, and it is still running. For labor income, we sum annual wages and other labor income for individuals who are household heads (male in married couple families, but female or male otherwise) and their partners; following e.g., Dynan, Elmendorf, and Sichel (2012), we obtain capital income as the difference between the reported total taxable income of the head and partner and the labor income calculated before, thus including dividends, interests, the part of business and farm income that accrues to business assets, rents, trusts, and royalties, and, when available, realized capital gains from the sale of assets. All income series are deflated into real 2017 US dollars using price levels provided by Feenstra, Inklaar, and Timmer (2015). For longitudinal consistency, we disregard the Latino sample, which was added only between 1990 and 1995.

To calculate the counterfactual income distributions, we obtain growth rates for the United States from Feenstra, Inklaar, and Timmer (2015). Following Piketty and Saez (2014) and Acemoglu and Robinson (2015), we define the growth rate of labor income $g_{t}$ as the sum of the population growth rate and the growth rate of per capita real GDP, while the real interest rate $r_{t}$ is obtained as the difference between the real internal rate of return and the average depreciation rate of the capital stock.^7^ In Section 4.2.3, we discuss a robustness analysis where the growth rate of national income is a weighted average of the growth rates of capital and labor income.

Because interviews in the PSID have been conducted biennially since 1997, we apply our Equation (3) to two-year changes in income (as in Dynan, Elmendorf, and Sichel 2012). Since income questions refer to the previous calendar year, and to take into account our need to calculate counterfactual distributions, the first two-year change in our sample is between 1969 and 1971 (from the 1970 and 1972 PSID waves). Then, the two-year changes overlap until 1996 (for example, the second two-year change is between 1970 and 1972), after which the two-year changes are not overlapping (e.g., the 1996–1994 difference is followed by the 1998–1996 one); given data from Feenstra, Inklaar, and Timmer (2015) are available only up to 2019, the last two-year change is between 2016 and 2018 (i.e., from the 2017 and the 2019 PSID waves).

Summary statistics are presented in Table 1; replication files are provided in Abatemarco, Lagomarsino, and Spiganti (2025).

### 4.2 | Results

**FIGURE 1 |:**
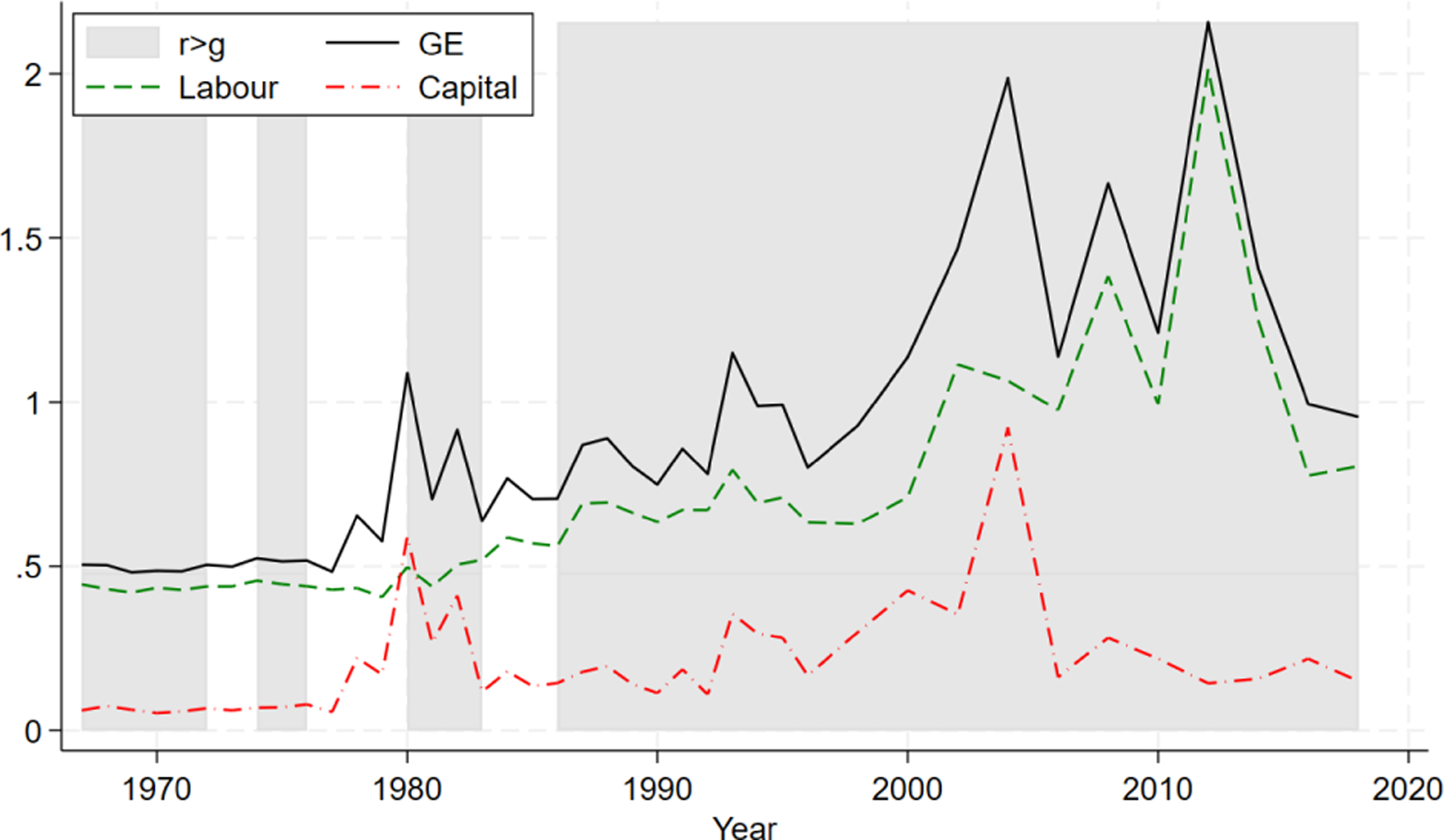
Inequality and contribution by income source.

We begin our analysis by examining the evolution of income inequality in the United States from 1967 to 2018. Overall inequality, quantified using the generalized entropy index GE defined in Equation (2), is represented by the solid line in Figure 1. Consistent with the findings of Piketty and Saez (2003) and Atkinson, Piketty, and Saez (2011), we observe a long-term upward trend in income inequality, with short-run fluctuations, primarily influenced by tax reforms, economic recessions, and the financial crisis.

Figure 1 also presents the decomposition of overall inequality following from Equation (4), which shows the contributions of labor and capital incomes separately. This reveals that labor income (dashed line) has consistently contributed more to overall inequality than capital income (dash-dotted line) throughout the period. The relative contributions of these two income sources have remained fairly stable, with labor income inequality accounting for approximately 80% of total inequality. Notable exceptions occurred following US tax reforms during the early 1980s and in 1993, and at the onset of the 2007 financial crisis, when the contributions of labor and capital to inequality were nearly equal.

#### 4.2.1 | The Piketty’s Component

**FIGURE 2 |:**
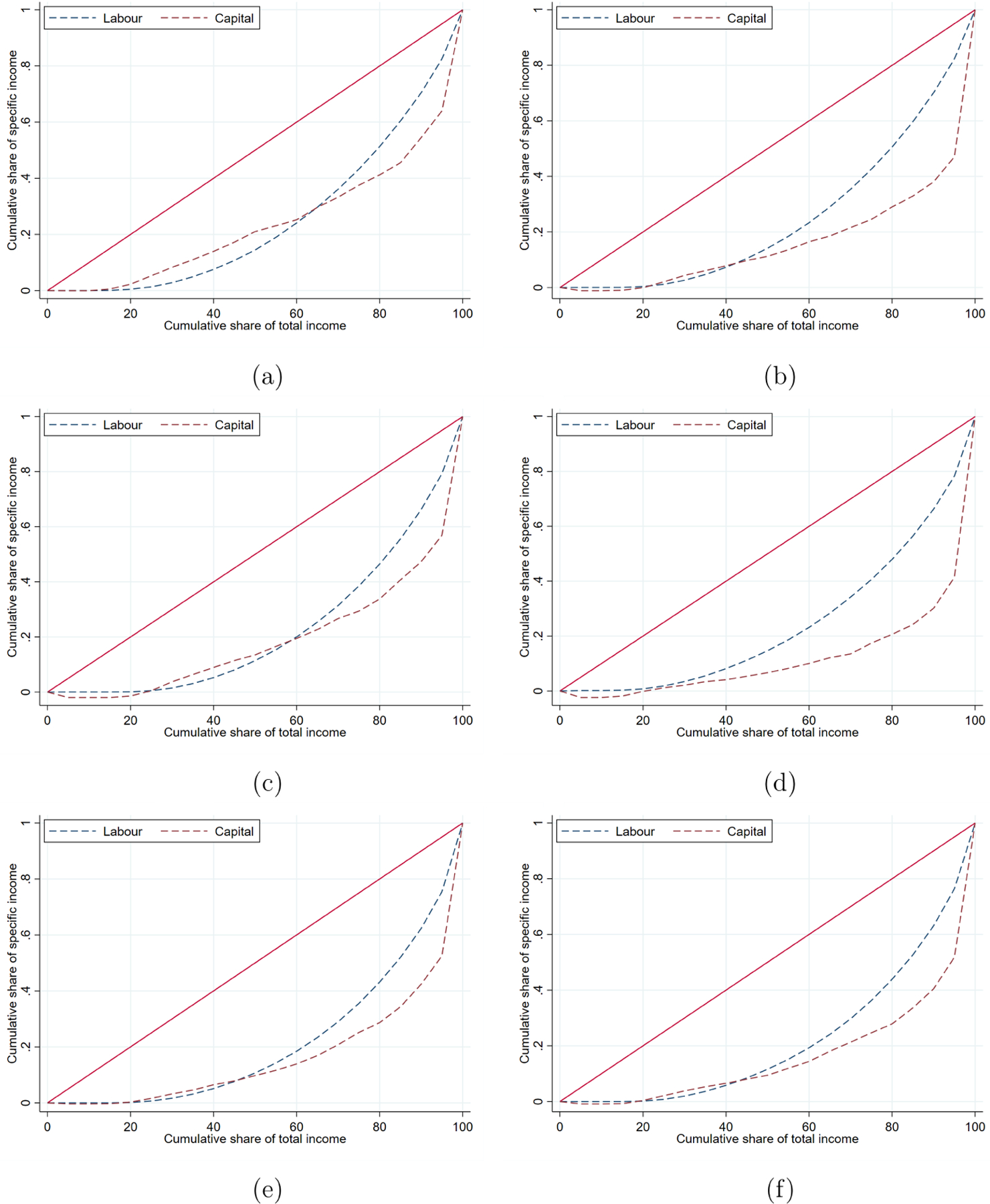
Concentration curves. (a) 1970. (b) 1980. (c) 1990. (d) 2000. (e) 2010. (f) 2018.

We now turn to the partial ordering analysis described in Section 3.1, which could be used to test for the existence condition of Piketty’s third law. Figure 2 provides an example, displaying the concentration curves of labor and capital incomes at the beginning of each decade considered and for the last year available. In all cases, we observe that the two concentration curves cross, preventing us from drawing a definitive conclusion regarding Piketty’s hypothesis. Nevertheless, the existence condition is (weakly) supported, as the concentration curve for labor income tends to lie above that of capital income.

Our complete ordering analysis based on the generalized entropy index allows for a more thorough investigation of the existence condition of Piketty’s third law. As explained in Section 3.2, if the existence condition holds, we should observe positive values of the Piketty’s component $\Delta GE_{t}^{P}$ during periods when $r_{t}>g_{t}$ and negative values when $r_{t}<g_{t}$. Here, we present two sets of results. The first set provides a short-term analysis examining the biennial evolution of $\Delta GE_{t}$ and its decomposition. The second one offers a medium- and long-term analysis, focusing on average changes in $\Delta GE_{t}$ over extended periods characterized by consistently positive or negative r-g gaps; we argue that this second analysis is more aligned with the spirit of Piketty’s (2014) original contribution.

**FIGURE 3 |:**
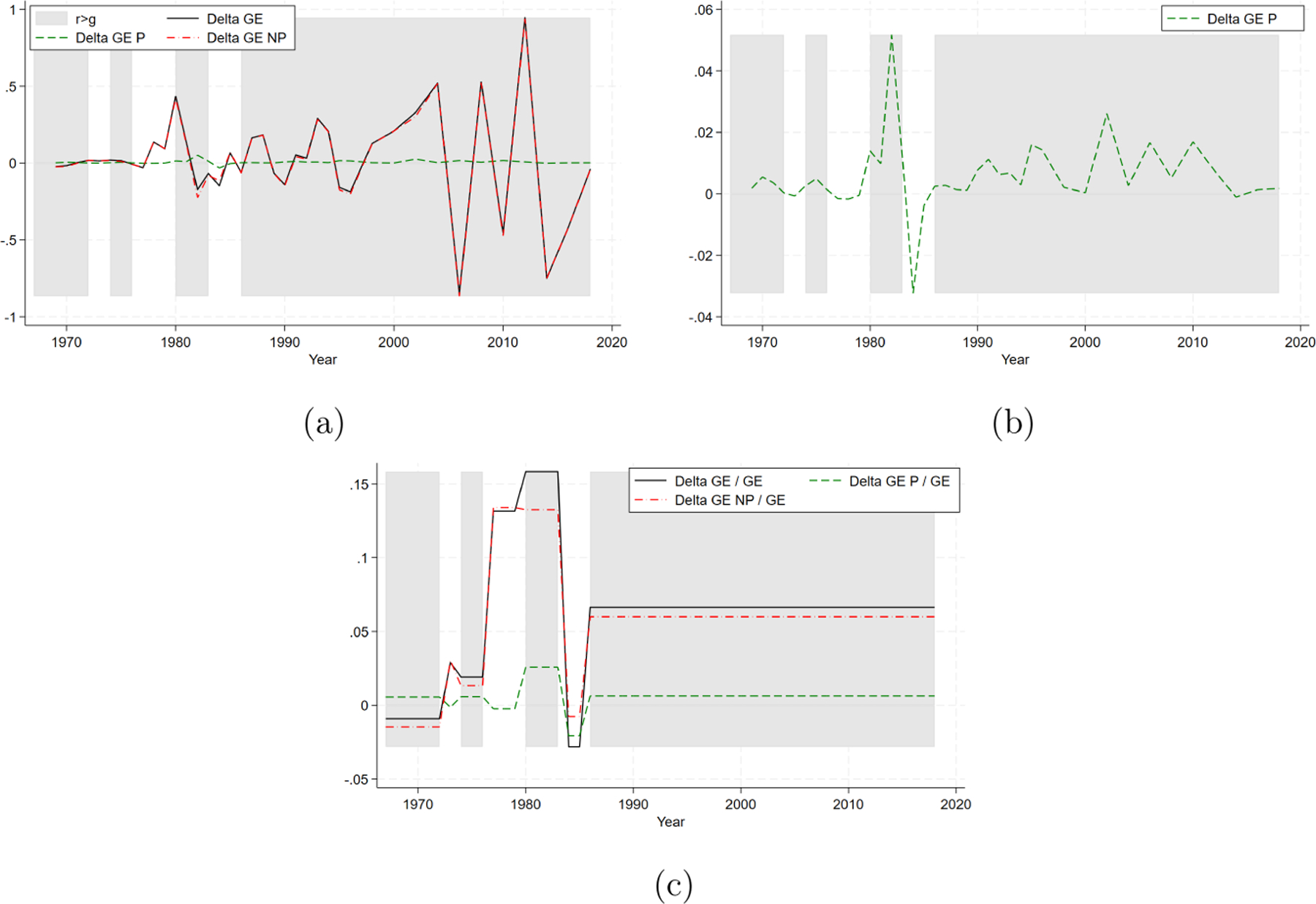
Piketty’s and non-Piketty’s components. (a) Variation of inequality over time. (b) Piketty’s component. (c) Average trends.

To aid comprehension, we present our results graphically. In the background of all figures, periods where r>g are shaded in grey, while periods with r<g are unshaded. Figure 3 represents the short-term analysis, showing the biennial evolution of $\Delta GE_{t}$ (solid line) along with its two components, $\Delta GE_{t}^{P}$ (dashed line) and $\Delta GE_{t}^{NP}$ (dash-dotted line), as derived from the decomposition IN Equation (3).

To verify the existence condition, it is essential to focus on the trend of Piketty’s component: given the relatively limited variation in this component shown in Figure 3, we zoom in on the $\Delta GE_{t}^{P}$ component in Figure 3. This closer examination of the short-term effects of Piketty’s component provides empirical support for the existence condition: we indeed observe that positive (cfr. negative) values of $\Delta GE_{t}^{P}$ align with periods when $r_{t}>g_{t}$ (cfr. $r_{t}<g_{t}$).

Focusing on the medium- and long-term, Table 2 provides the average biennial growth rates of overall income inequality $GE_{t}$ (third column) and its decomposition into average Piketty’s (fourth column) and non-Piketty’s (fifth column) components, across time windows with consistent r-g sign. Figure 3c displays these average trends. The fourth column of Table 2, or equivalently the dashed line in Figure 3c, provides strong support for the verification of the existence condition. As already observed in Figure 3b for the short run, there is indeed a perfect correspondence between the sign of the r-g gap (second column) and the sign of the average $\Delta GE_{t+k}^{P}/GE_{t}$ in each of the seven time windows. Specifically, periods with r>g show a positive sign and periods with r<g show a negative sign. Notably, this correspondence does not hold when considering the average growth rate in overall inequality (third column, or solid line in Figure 3c).

We now turn to the quantification of the relative importance of the Piketty’s component. Figure 3a shows that the non-Piketty’s component $\Delta GE_{t}^{NP}$ dominates in the short term, with the solid $\left(\Delta GE_{t}\right)$ and dash-dotted $\left(\Delta GE_{t}^{NP}\right)$ lines nearly overlapping across all periods. This suggests that factors beyond the r-g mechanism are almost entirely responsible for driving overall income inequality over the short term. Indeed, the Piketty’s component contributes only marginally to the trend in overall income inequality over the short-run: on average, its relative importance (i.e., the average $\Delta GE_{t+k}^{P}/\Delta GE_{t+k}$) is approximately 2.81%, but this is almost entirely driven by the first decade, characterized by relatively constant inequality levels. As evident from Figure 3a, the variation in overall inequality is very volatile in the short term, especially in more recent decades, and the Piketty’s component is too smooth to explain it.

Conversely, the values presented in Table 2 (and illustrated in Figure 3c) show that the Piketty’s component has a more substantial impact in the medium- and long-term. For instance, in the longest window (the last 32-year period), the Piketty’s component accounts for 0.64 percentage points of 6.64% points of overall inequality variation, that is, for approximately 9.65% of the overall trend. Similarly, the average biennial growth rates of overall inequality, the Piketty’s component, and the non-Piketty’s component over the entire span of our data (i.e., the weighted average of column three, four, and five of Table 2, respectively, where the weights are the relative lengths of each period) are approximately 6.37%, 0.60%, and 5.76%. The Piketty’s component thus accounts on average for 0.60 of 6.37% points, that is, for approximately 9.45% of the growth rate of overall income inequality in the long run. Therefore, the contribution of $\Delta GE_{t}^{NP}$ to the overall inequality trend is consistently larger than that of $\Delta GE_{t}^{P}$ in nearly all periods (and on average) also in the medium- and long-term, often determining the direction of the overall trend when the two components have opposite signs.

#### 4.2.2 | The Non-Piketty’s Component

**FIGURE 4 |:**
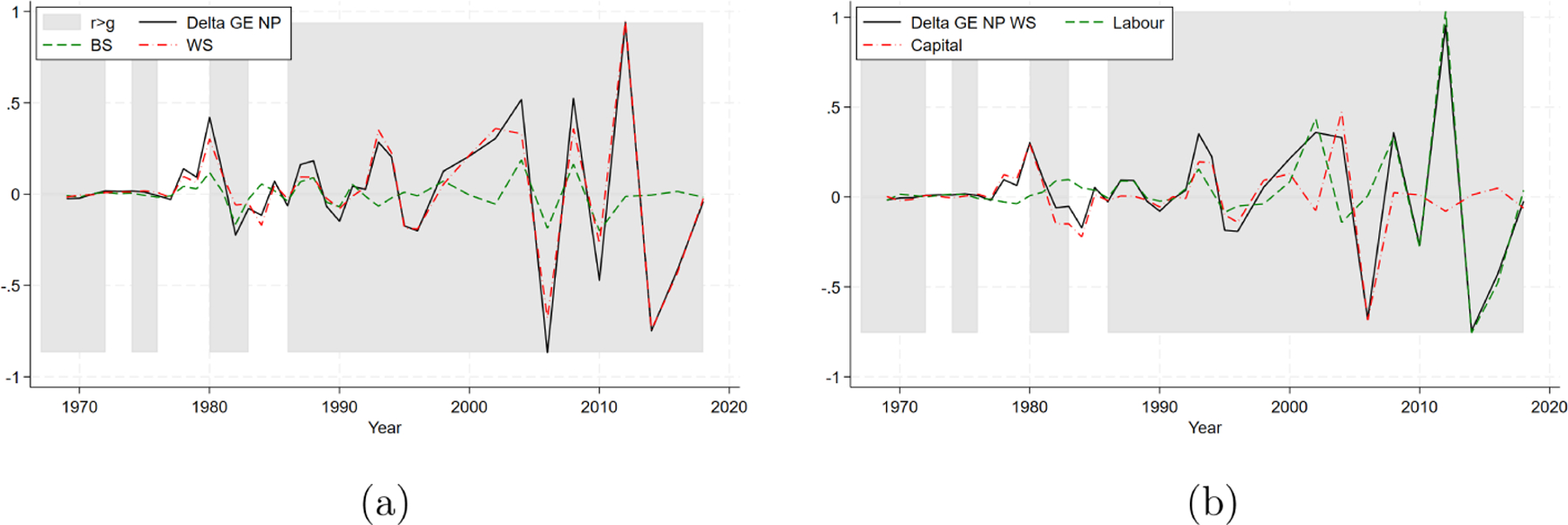
Non-Piketty’s component. (a) Between- and within-source. (b)Within-source.

Having examined the existence condition for Piketty’s third law and its relative importance, we shift our focus to analyzing the non-Piketty’s component of the inequality trend, as it is the predominant contributor to inequality variation in both the short term and in long term. In particular, Figure 4a presents $\Delta GE_{t}^{NP}$ (solid line) and its two components, one stemming from between-source inequality ($\Delta {\hat{BS}}^{NP}$, dashed line) and one from within-source inequality ($\Delta {\hat{WS}}^{NP}$, dash-dotted line), following the decomposition outlined in Equation (6). This indicates that income inequality dynamics, net of Piketty’s effects, are primarily determined by changes in the within-source component (i.e., by the evolution of labor and capital income distributions taken separately).

Figure 4b then further decomposes the within-source component (net of Piketty’s effects) into the contributions of the individual income source distributions, that is, labor ($\Delta {\hat{WS}}^{NP(w)}$, dashed line) and capital ($\Delta {\hat{WS}}^{NP(c)}$, dash-dotted line), using the procedure outlined in Equation (7). Up to the financial crisis, changes in labor income inequality are generally smoother compared to changes in capital income inequality, which is more volatile, especially around periods of financial instability (as evident from Figure 1). However, the sharp changes in total inequality in the latest decade are mostly due to changes in labor income inequality.

#### 4.2.3 | Robustness Checks

In this section, we present two robustness checks concerning the way in which the counterfactual income distributions are constructed: the first one regards the use of an indirect (rather than direct) approach, whereas the second one uses an alternative growth rate for labor income.

First, the (direct) decomposition strategy outlined in Equation (3) can alternatively (though not equivalently) be reformulated by considering an indirect approach.^8^ In particular, let ${\overset{ˆ}{Y}}_{t|t+k}≔\left\{{\overset{ˆ}{y}}_{1,t|t+k},\ldots ,{\overset{ˆ}{y}}_{n_{t|t+k},t|+k}\right\}\in R_{\geq 0}^{n_{t|t+k}}$ represent the *counterfactual* income distribution at time t, obtained by removing the expected effect of Piketty’s income dynamics from the factual distribution at time t+k, that is

(8) $${\overset{ˆ}{y}}_{i,t|t+k}≔\frac{y_{i,t+k}^{w}}{1+g_{t}}+\frac{y_{i,t+k}^{c}}{1+r_{t}}\;\forall i$$

Using this *indirect counterfactual*, the variation of overall income inequality for any pair of consecutive periods can be then decomposed as

(9) $$\Delta GE_{t+k}=GE_{t+k}-GE_{t}\;=\left(GE_{t+k}-{\hat{GE}}_{t|t+k}\right)+\left({\hat{GE}}_{t|t+k}-GE_{t}\right)\;=\Delta GE_{t+k}^{P}+\Delta GE_{t+k}^{NP}$$

where ${\hat{GE}}_{t|t+k}$ measures inequality in the counterfactual distribution ${\overset{ˆ}{Y}}_{t|t+k}$. Here, $\Delta GE_{t+k}^{P}$ measures the expected effect of Piketty’s income dynamics, whereas $\Delta GE_{t+k}^{NP}$ represents its complement and accounts for all other drivers of inequality. In other words, in the direct decomposition approach, the Piketty’s component is derived by simulating labor and capital income growth at their respective rates, while the non-Piketty’s component is defined residually; this implies that any interaction between the two components is included in the non-Piketty’s component, ensuring that the Piketty’s component remains unaffected by such interaction. Conversely, in the indirect strategy, the Piketty’s component is obtained by solely eliminating Piketty’s dynamics without considering the potential impact of this elimination on other drivers. Consequently, any interaction between the two components remains solely within Piketty’s component.

**FIGURE 5 |:**
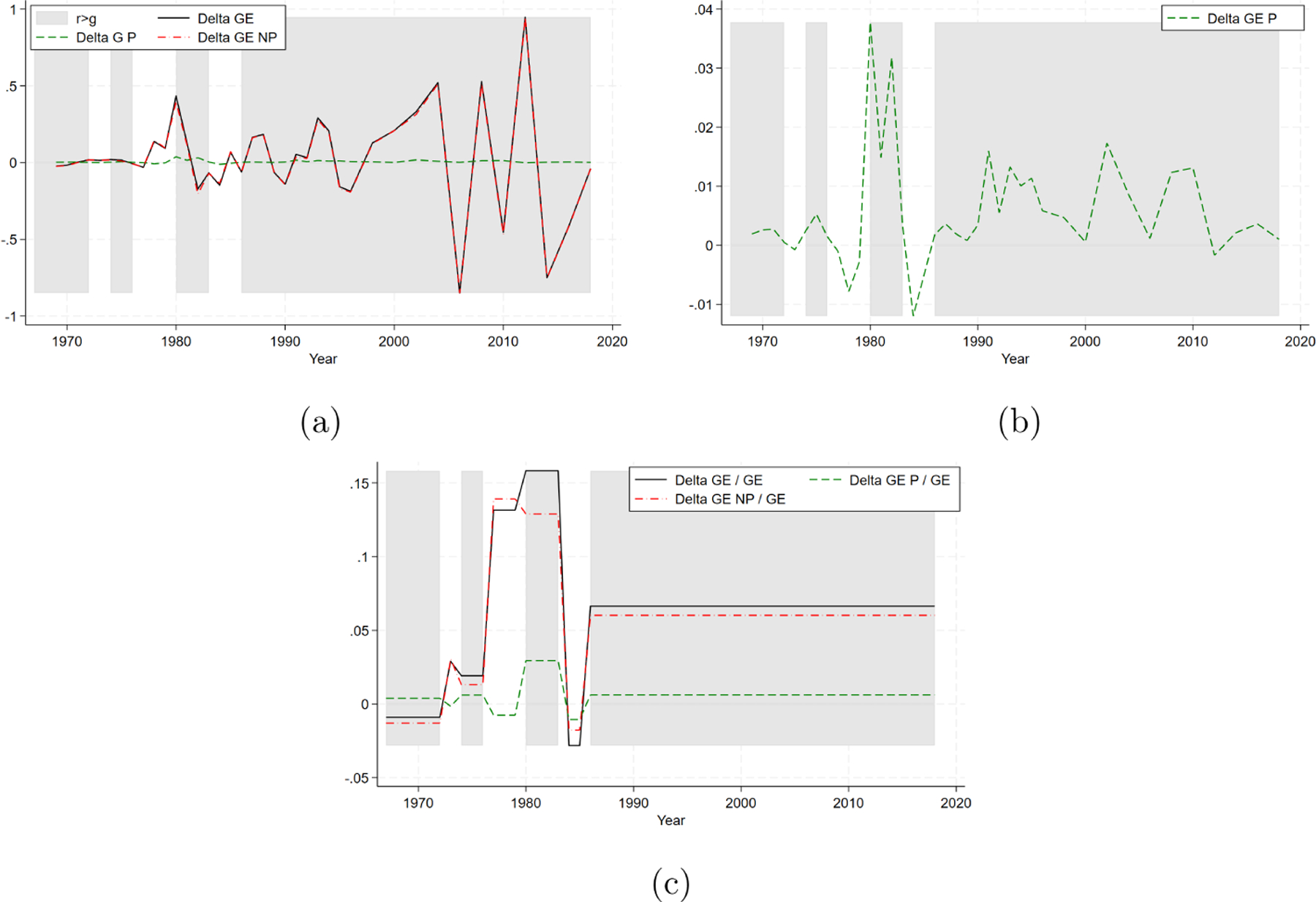
Indirect decomposition. (a) Variation of inequality over time. (b) Piketty’s components. (c) Average trends.

In Figure 5, we present the indirect versions of Figures 3a–c, which exhibit the same patters and approximately the same values compared to those obtained from the direct decomposition strategy (e.g., 9.76% of the average growth rate in overall income inequality is attributable to the Piketty’s component). This finding reinforces our results: the impact of Piketty’s income dynamics remains consistent over time and across approaches, albeit still relatively smaller compared to other drivers (and marginal in the short run). Since the disparity between the direct and indirect approaches stems from the interaction between the Piketty’s component and the non-Piketty’s component, this disparity is expected to be minimal when the Piketty’s and non-Piketty’s components are independently distributed, which appears to be the case here.

Second, in the empirical analysis, we defined the growth rate of labor income $g_{t}$ as the sum of the population growth rate and the growth rate of per capita real GDP, which we consider in line with Piketty (2014) and Acemoglu and Robinson (2015). However, if national income is the sum of labor income and capital income, then the growth rate of national income $g_{t}$ is actually a weighted average of the growth rate of capital income $r_{t}$ and the growth rate of labor income $n_{t}$, with weights given by factor shares. As a consequence, the growth rate of labor income can be expressed as $n_{t}=\left(g_{t}-\alpha_{t}r_{t}\right)/\left(1-\alpha_{t}\right)$, where $1-\alpha_{t}$ is the labor share, which is available from Feenstra, Inklaar, and Timmer (2015).

**FIGURE 6 |:**
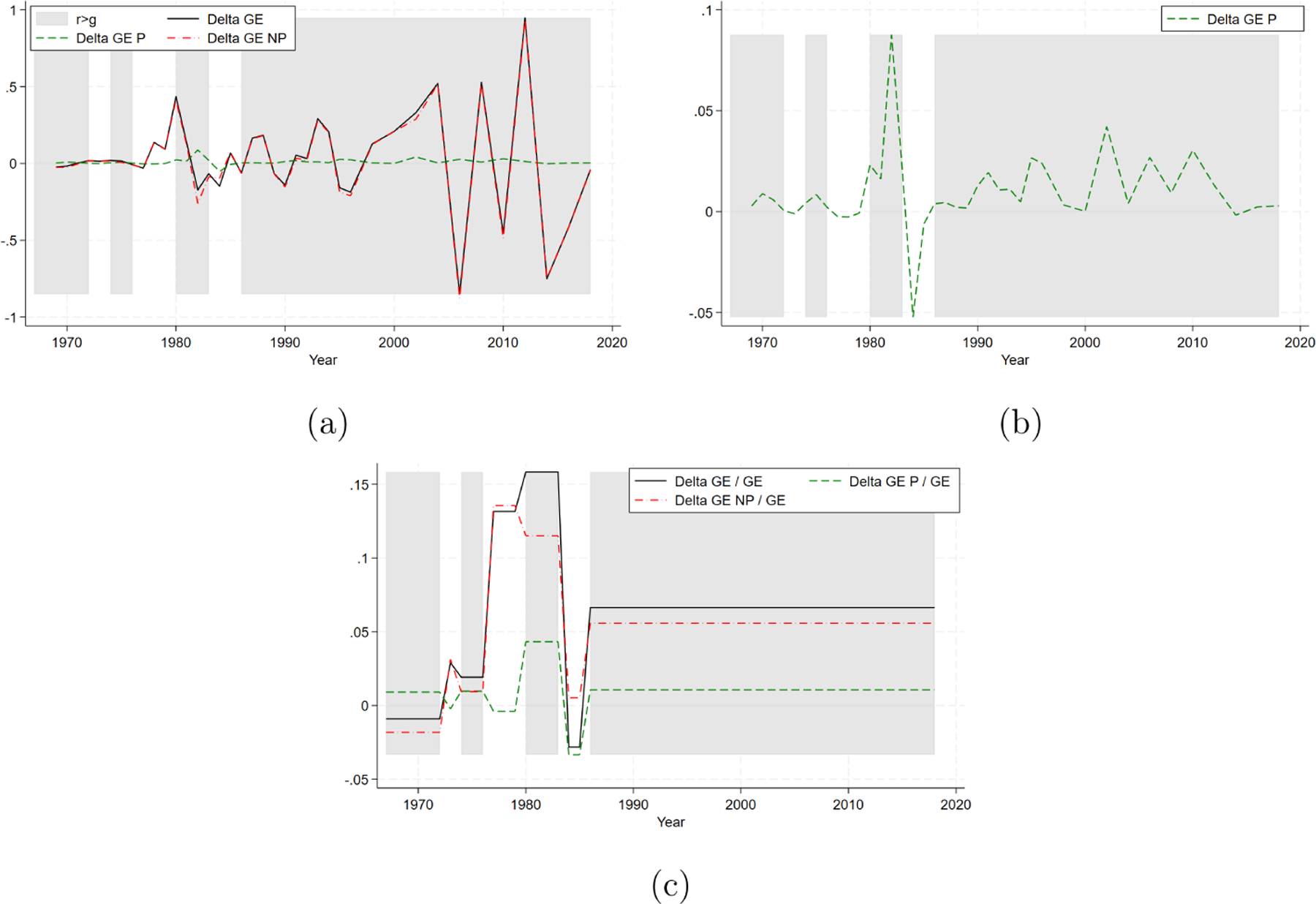
Alternative growth rate of labor income. (a) Variation of inequality over time. (b) Piketty’s components. (c) Average trends.

Figure 6 shows the alternative versions of Figures 3a–c, obtained using $n_{t}$ (rather than $g_{t}$) to calculate the counterfactual labor income distributions. These exhibit the same patters as those in Section 4.2, but with a greater relative importance of the Piketty’s component; for example, this component explains approximately 15.77% of the average biennial growth rate of overall income inequality over the entire period. However, given our previous results, this is to be expected. Indeed, $n_{t}$ is lower (cf. higher) than $g_{t}$ when $g_{t}<r_{t}$ (cf. $g_{t}>r_{t}$): using $n_{t}$ to calculate the counterfactual distributions then increases the importance of the Piketty’s component if capital income is mostly held by top income classes, which is the case in the PSID.

## 5 | Concluding Remarks

In this paper, we proposed a novel non-parametric measurement strategy to decompose inequality changes over time between a Piketty’s component, capturing inequality changes due to capital and labor income growing at different rates, and a non-Piketty’s component, due to any other driver of inequality, such as distributional shifts. We use this strategy to test Piketty’s third law, positing that a positive r-g gap will tend to be associated with an increase in income inequality.

Analyzing the relationship between the gap in the rate of return to capital r and economic growth g and income inequality in the United States using data from the PSID for the last five decades, we obtained three main sets of results. First, we found evidence in support of the existence of Piketty’s third law, both in the short term and in the long term. Second, our findings indicated that factors other than Piketty’s component predominantly drive variations in overall income inequality. In the short term, the Piketty’s component is smooth and thus unable to explain the very volatile change in overall income inequality; however, in the medium- and long-term, approximately 10% of the average increase in overall income inequality is attributable to the Piketty’s component. As this effect is not negligible, we argue that the Piketty’s component should be opportunely considered among the determinants of inequality in the medium- and long-term. Third, we highlighted that within-source inequality, particularly driven by labor income, remains a critical determinant of overall income inequality.

Whereas we studied the evolution of income inequality in the United States for the last 50 years, our methodological approach can be applied to microeconomic data from different countries and time periods. This would be particularly interesting given the suggestions that top income shares have evolved differently in Anglo-Saxon countries than in continental Europe (Atkinson and Piketty 2007) and that whether a rising capital’s share of national income leads to greater inequality may vary across different social systems, institutional contexts, and production technologies (Acemoglu and Robinson 2015; Milanović 2017; Bengtsson and Waldenström 2018; Ranaldi and Milanović 2022). We leave these applications for future research.

## Figures and Tables

**TABLE 1 |:** Summary statistics.

			Average income			Standard deviation		
Year	g	r	Labor	Capital	Total	Labor	Capital	Total
1967	9.35%	13.32%	30,989	2,411	33,400	30,696	9,315	33,568
1968	7.66%	12.73%	32,469	2,699	35,168	31,626	10,908	35,297
1969	8.09%	11.98%	33,829	2,600	36,429	32,626	10,811	35,768
1970	3.33%	11.16%	33,802	2,470	36,272	33,061	9,431	35,784
1971	3.47%	9.88%	33,828	2,491	36,319	32,744	9,815	35,766
1972	8.60%	9.46%	34,969	2,620	37,589	34,233	11,090	37,768
1973	11.07%	9.51%	36,110	2,683	38,792	35,387	10,799	38,751
1974	5.05%	9.25%	35,547	2,662	38,208	35,381	11,068	39,127
1975	−0.69%	8.25%	34,347	2,622	36,969	34,067	11,657	37,514
1976	5.12%	7.27%	35,983	3,108	39,091	35,822	13,526	39,767
1977	10.12%	7.52%	37,226	2,930	40,156	36,515	11,505	39,500
1978	10.27%	7.76%	38,507	3,489	41,996	37,254	25,297	48,044
1979	8.74%	7.53%	39,319	3,549	42,868	37,216	22,798	46,007
1980	2.83%	7.08%	38,590	4,273	42,863	38,234	42,443	63,246
1981	2.17%	6.19%	37,989	4,052	42,040	37,662	28,562	49,904
1982	0.55%	5.86%	37,098	3,900	40,998	40,309	36,124	55,484
1983	2.50%	5.62%	38,063	3,889	41,952	41,775	18,233	47,380
1984	11.81%	6.10%	40,257	4,101	44,357	46,039	22,726	54,964
1985	11.43%	7.80%	41,441	3,959	45,400	47,076	20,604	53,906
1986	7.59%	8.79%	42,475	4,152	46,626	48,066	22,271	55,405
1987	6.86%	8.85%	43,915	4,347	48,262	53,947	22,805	63,649
1988	7.58%	8.49%	45,853	4,683	50,536	56,220	24,766	67,406
1989	7.76%	8.38%	41,705	3,972	45,676	50,789	20,022	57,927
1990	5.40%	8.96%	42,059	3,742	45,801	49,832	17,310	56,051
1991	1.64%	9.41%	41,183	3,717	44,900	49,082	21,197	58,816
1992	3.28%	9.25%	43,574	3,480	47,054	52,920	17,674	58,800
1993	6.23%	9.34%	46,467	5,410	51,876	63,557	41,026	78,656
1994	6.78%	9.74%	46,669	5,632	52,300	60,252	38,090	73,499
1995	6.71%	10.28%	48,081	5,632	53,712	61,700	36,613	75,632
1996	6.46%	10.42%	50,236	5,879	56,115	61,880	29,900	71,006
1998	8.97%	10.35%	55,193	7,425	62,618	66,736	43,183	85,315
2000	8.91%	9.01%	59,856	7,643	67,499	77,089	57,853	101,774
2002	2.70%	7.14%	58,530	6,162	64,692	95,124	51,727	110,824
2004	6.67%	7.34%	59,141	7,368	66,509	91,076	83,898	132,614
2006	6.38%	7.63%	59,225	6,475	65,700	90,131	33,200	99,126
2008	1.71%	6.80%	59,810	5,906	65,716	104,570	37,570	119,950
2010	−0.01%	6.25%	53,987	4,981	58,967	82,131	36,753	91,781
2012	3.80%	7.12%	54,735	4,651	59,387	118,677	29,835	123,378
2014	4.37%	6.70%	53,894	4,448	58,342	91,693	31,131	97,896
2016	4.79%	6.18%	55,781	4,773	60,554	74,317	37,835	85,375
2018	5.35%	6.01%	57,633	4,446	62,079	78,034	32,368	85,778

*Note:* Income values are own elaborations from the Panel study of income dynamics (2024). Growth rates are from Feenstra, Inklaar, and Timmer (2015) and refer to two-year changes in the United States.

**TABLE 2 |:** Average period effects.

Period	r-g	$\Delta GE_{t+k}/GE_{t}$	$\Delta GE_{t+k}^{P}/GE_{t}$	$\Delta GE_{t+k}^{NP/GE_{t}}$
1967–1972	+	−0.91%	0.57%	−1.47%
1973	−	2.90%	−0.14%	3.04%
1974–1976	+	1.92%	0.59%	1.33%
1977–1979	−	13.16%	−0.24%	13.40%
1980–1983	+	15.83%	2.58%	13.25%
1984–1985	−	−2.82%	−2.06%	−0.76%
1986–2018	+	6.64%	0.64%	6.00%

## Data Availability

The data that support the findings of this study are available in NA at [https://doi.org/10.3886/E215161V1]. These data were derived from the following resources available in the public domain:- Panel study of income dynamics, https://psidonline.isr.umich.edu/- Penn World Table, https://www.rug.nl/ggdc/productivity/pwt/?lang=en.

## Footnotes

1 Similarly to ours, these articles focus on income inequality. However, other studies have reached different conclusions examining wealth inequality. For example Madsen, Minniti, and Venturini (2018), use data from OECD countries since 1870 and finds that the wealth-to-income ratio is negatively related to g. Using administrative data from Norway between 2010 and 2018 Iacono and Palagi (2023), document that r-g differs across the net wealth distribution: since this is higher (cf. lower) than the average for the top 40% (cf. bottom 60%), the distribution of the r-g gap predicts a higher level of income and wealth inequality than the aggregate gap. Finally, both Fuest, Peichl, and Waldenström (2015), using cross-country panel data, and Strauss and Ventosa-Santaularia (2023), using wealth shares for different percentiles for the United States, find a positive relationship between r-g and the evolution of wealth inequality.

2 See also Ranaldi and Milanović (2022) and Iacono and Ranaldi (2023), who instead study homoploutia for different countries using the income factor concentration index developed in Ranaldi (2022).

3 Let $L_{t}(p)$ and ${\overset{ˆ}{L}}_{t+k|t}(p)$ indicate the Lorenz curves of, respectively, the income distribution at time t and the counterfactual distribution one would observe at t+k were Piketty’s income dynamics the only transformation operating from t to t+k; also, let $C^{s}(p)$ be the concentration curve of $Y_{t}^{s}$, which shows how income from labor (s=w) or capital (s=c) is distributed across the population ranked according to total income. Given our definition of ${\overset{ˆ}{Y}}_{t+k|t}$, the following equivalence conditions must hold by construction. $L_{t}\left(p\right)⋚{\overset{ˆ}{L}}_{t+k|t}\left(p\right)\;\forall \;p,r_{t}>g_{t}\Leftrightarrow C_{t}^{w}(p)⋚C_{t}^{c}(p)$ and $L_{t}\left(p\right)⋛{\overset{ˆ}{L}}_{t+k|t}\left(p\right)\;\forall \;p,r_{t}<g_{t}\Leftrightarrow C_{t}^{w}\left(p\right)⋚C_{t}^{c}\left(p\right)\;\forall \;p$.

4 The generalized class of entropy measures GE(a) is defined as $[na(a-1){]}^{-1}\sum_{i=1}^{n}\left[{\left(y_{i}/\mu_{Y}\right)}^{a}-1\right]$ for real a different from 0 and 1, $n^{-1}\sum_{i=1}^{n}\left[\left(y_{i}/\mu_{Y}\right)\;\text{ln}\left(y_{i}/\mu_{Y}\right)\right]$ for a=1, and $-n^{-1}\sum_{i=1}^{n}\left[\text{ln}\left(y_{i}/\mu_{Y}\right)\right]$ for a=0, where a is a parameter regulating the weight given to distances between incomes at different parts of the earnings distribution (the greater is a, the more the index is sensitive to high incomes). As far as GE indices have a quasi-separable form, decomposability by income source holds for all values of a (Shorrocks 1982). However, if one also requires the two-factor symmetry condition to hold (whereby two income sources are assigned the same contribution to inequality if the distribution of income from both sources is identical and if together they make up total income), then decomposability by income sources is satisfied at a=2 only.

5 As discussed in Section 4.2.3, an indirect version of this decomposition strategy can be obtained by discounting the r and g factors from the observed income distribution at time t+k.

6 Conversely, the decomposition by sources of the Gini index would not allow us to exploit this property because covariances are defined with respect to rankings in the income distribution.

7 Using the same interest rate and growth rate for the entire distribution of income may underestimate (cfr. overestimate) the true r-g for the top (cfr. bottom) of the distribution (Iacono and Palagi 2023).

8 The distinction between direct and indirect measurement strategies is very common in the application of the counterfactual approach to inequality decomposition (e.g. Wagstaff and van Doorslaer 2000; Ramos and Van de Gaer 2016).
